# Clinical monitoring of activated clotting time during cardiothoracic surgery: comparing the Hemochron^®^ Response and Hemochron^®^ Signature Elite

**DOI:** 10.1177/02676591211049316

**Published:** 2021-10-01

**Authors:** Roland F Hoffmann, Sandra Horsten, Massimo A Mariani, Adrianus J de Vries

**Affiliations:** 1 Department of Cardiothoracic Surgery, Section Extracorporeal Circulation, University of Groningen, University Medical Center Groningen, Groningen, The Netherlands; 2 Department of Cardiothoracic Surgery, Section Thoracic Surgery, University of Groningen, University Medical Center Groningen, Groningen, The Netherlands; 3 Department of Anesthesiology, University of Groningen, University Medical Center Groningen, Groningen, The Netherlands

**Keywords:** activated clotting time, cardiothoracic surgery, heparin, cardiopulmonary bypass, bias, variability

## Abstract

**Introduction:**

The Activated Clotting Time (ACT) is commonly used to manage anticoagulation during cardiac surgery. The aim of this study was to compare the older manually operated Hemochron^®^ Response and the automated Hemochron^®^ Signature Elite.

**Methods:**

In this observational study the clinically relevant differences of both devices were investigated simultaneously, using duplicate measurements, in 29 patients who underwent a Coronary Artery Bypass Grafting (CABG) or Aortic Valve Replacement (AVR) in order to determine reliability, bias, and to detect which method has the lowest variation. Blood samples were obtained from the arterial line prior to surgery, after administration of 300 IU/kg heparin, 5 minutes after initiation of cardiopulmonary bypass and successively every 30 minutes, and after protamine administration.

**Results:**

A total of 202 measurements were performed. Of these 10 measurements were out of range in the Response and 9 in the Elite. About 27 single unstable magnet errors were seen in the Response versus no measurement errors in the Elite. No statistically significant differences between the Response (p = 0.22, Wilcoxon rank) and Elite (p = 0.064) duplicates were observed. The Response values were consistently higher during heparinization than the Elite measurements (p = 0.002, repeated measurements) with an average positive bias of around 56 seconds during heparinization (Bland-Altman). Overall, the coefficient of variation (CoV) increased during heparinization.

**Conclusion:**

The Elite was more reliable, but the variation was higher for the Elite than the Response. The observed positive bias in the Response compared to the Elite could affect heparin administration during surgery making the two systems not interchangeable.

## Introduction

Heparin is one of the most frequently administered drugs during cardiothoracic surgery using Cardiopulmonary Bypass (CPB). In clinical practice, adequate heparin management is pivotal to prevent coagulation but also to prevent excessive blood loss after surgery.^1,2^ The Activated Clotting Time (ACT) is commonly used to manage anticoagulation and to control heparin dosage for each patient. Even though the ACT has been in use since the late sixties/early seventies, it still remains the gold standard to measure the effect of heparin during CPB.^3–6^ This strategy, to monitor the heparin dosage for each patient based on the ACT, has proven to be superior over fixed heparin dosage schemes on postoperative blood loss.^7^ ACT values greater than 400 or 480 seconds are generally maintained during CPB.^8^

In 2005, a relatively new device, the Hemochron^®^ Signature Elite (International Technidyne Corporation, Edison, NJ, USA) has become available for use during cardiothoracic surgery. The Hemochron^®^ Signature Elite, as well as the Hemochron^®^ Jr Signature Plus, are updated versions of the Hemochron^®^ Jr with improved software options but the same clot detection system.^9^ The system comes with low and high range ACT cuvettes and uses a mixture of silica, kaolin, and phospholipids as activator to initiate coagulation, which is thought to be a faster and more effective alternative to existing ACT tests that use only either celite or kaolin as activator.^10,11^ Because the test is fully automated it is expected to produce more precise results compared to the older manually operated systems where the operator has to inject the right amount of blood into a test tube.^12,13^ Despite widespread clinical use of the Hemochron® Jr ACT systems there are only two studies available that compare bias and variability in high heparin dosages such as in cardiac surgery between this system and older devices.^14,15^

The aim of this study was to compare reliability, bias and variation in ACT measurements between the Hemochron® Response and the Hemochron^®^ Signature Elite in patients undergoing cardiothoracic surgery.

## Methods

Adult patients undergoing Coronary Artery Bypass Grafting (CABG) or Aortic Valve Replacement (AVR) with CPB were eligible for inclusion in this observational study. Exclusion criteria were known coagulopathy and patients on continuous heparin therapy. All participants gave written informed consent to this study which was approved by the ethics committee of the University Medical Center Groningen (METc 2007/124).

For the ACT measurements one Hemochron® Response device (International Technidyne Corporation, Edison, NJ, USA) which has two channels, and two (randomly picked from a pool of 5) Hemochron^®^ Signature Elite devices were used. For the Hemochron® Response measurement the operator injects 2 ml of blood into the Celite ACT test tubes. It is mixed with the activator and clot formation is detected when the magnet in the test tube is displaced. With the Hemochron^®^ Signature Elite blood is automatically drawn into the cuvette and mixed with the activators (containing kaolin, silica, and phospholipids). The clotting time is calculated based on optical analysis of the speed at which the 0.15 ml blood sample moves between the sensors. The devices were properly serviced by the company prior to the study and were used according to manufacturer’s protocol. Both devices were pre-warmed before use and the test tubes (containing 12 mg of celite) for the Hemochron^®^ Response and Hemochron^®^ Jr ACT+ cuvettes for the Hemochron^®^ Signature Elite were kept at room temperature prior to use. One of the Hemochron^®^ Signature Elite devices was in the operating room and was used to guide anticoagulation. The Hemochron^®^ Response and the other Hemochron^®^ Signature Elite were outside the operating room, and the personnel caring for the patient were blinded to the readings.

Blood samples were collected from the arterial line (without heparin flush) using 5 ml syringes or from the sample line of the CPB system during bypass to determine the ACT at baseline (*T*1), after administration of 300 IU/kg heparin (LEO Pharma, The Netherlands) (*T*2), 5 minutes after initiation of CPB (*T*3), 30 minutes after initiation of CPB (*T*4), 60 minutes after initiation of CPB (*T*5), 90 minutes after initiation of CPB (*T*6), 120 minutes after initiation of CPB (*T*7), and after protamine administration (*T*8). The number of blood samples was limited to eight per individual. Protamine was administered in a 1:1 ratio to the initial dose of heparin as per institutional protocol. The baseline sample after administration of heparin and final sample after administration of protamine were taken 5 minutes after heparin or protamine administration. When a measurement reached 1000 seconds, the measurement was stopped and 1000 seconds was used. An out-of-range measurement was noted accordingly.

The CPB circuit consisted of a centrifugal pump (Revolution, LivaNova, United Kingdom) with a flow of 2.4 L/m^2^/minutes, an oxygenator (Inspire 8L, LivaNova, United Kingdom) and cardiotomy reservoir (Inspire, LivaNova, United Kingdom). The circuit was primed with 1200 ml lactated Ringer’s solution (Baxter B.V., The Netherlands), 500 ml hydroxyethyl starch 6% (Voluven®, Fresenius Kabi, Germany), and 5000 IU of heparin. Depending on the surgical procedure 2 or 3 machine suckers were used. Hemodilution was monitored for all patients by comparing the Hemoglobin (Hb) 5 minutes after the start of CPB (i.e. before administration of cardioplegia) to the preoperative Hb. Temperature was allowed to drift to 35°C. All patients received 2 g of tranexamic acid before initiation of CPB.

### Statistical analysis

The sample size was estimated based on the ACT values measured using the Hemochron® Response from our previous study.^16^ There we found a mean ACT of 510 seconds with a standard deviation of 133 seconds. We estimated that the Hemochron^®^ Signature Elite had about 8% lower readings. With the usual assumptions of beta 0.2 and alpha 0.05 this would require 174 comparisons, which would require at least 27 patients at an average of 6.4 measurements per patient.

Normal distribution was tested using the Shapiro-Wilk test. Non-parametric data was analyzed by a Friedman ANOVA, Wilcoxon signed rank test (pairwise), Kruskal-Wallis ANOVA or the Mann-Whitney-*U* test to determine statistical significance within or between groups respectively. A Wilcoxon signed rank test was used for pairwise comparison of the two methods over time. Dispersion was calculated with the coefficient of variation. Bland-Altman plots were used to visualize bias and precision. SPSS (version 26) was used for the analysis. Normally distributed data is expressed as mean ± standard deviation (SD). Non-normally distributed data is expressed as median [interquartile range (IQR)]. Differences were considered statistically significant at a p-value of less than 0.05. The null-hypothesis assumed equal measures.

## Results

A total of 37 patients were included in this study between July 2017 and May 2018. For logistic reasons such as postponed surgery, measurements could not be obtained in eight patients. Thus, 29 patients completed the study. The baseline demographic data are presented in Table 1.

**Table 1.** Demographic and procedure data (*n* = 29).

Age (years)	68 ± 9
Male (*n*, %)	23 (79)
Weight (kg)	90 ± 12.7
Height (cm)	175 ± 9
BSA (m^2^)	2.1 ± 0.2
Diabetes (*n*, %)	10 (34)
Preoperative hemoglobin (g/dl)	14.5 ± 1.0
Preoperative platelet count (×10^9^/l)	248 ± 63
CABG (*n*, %)	23 (79)
AVR (*n*, %)	6 (21)
CPB time (minutes)	107 ± 24
Pump flow (L/minutes)	5.1 ± 0.5
Hemoglobin on bypass (g/dl)	9.7 ± 1.1
Hemodilution (%)	28 ± 4
Total heparin (IU)	40,517 ± 10,905

Data are mean ± SD.

BSA: body surface area; CABG: coronary artery bypass grafting; AVR: aortic valve replacement; CPB: cardiopulmonary bypass.

For each patient measurements were collected for *T*1–*T*8, and at each time point four ACT values were measured. However, not all time points could be collected for every patient because that depended on the duration of the bypass. Therefore, at 90 minutes CPB (*T*6) there were 20 patients, and at 120 minutes CPB (*T*7) there were only 8 patients. Thus, 202 duplicate measurements were performed in total.

Occasionally values greater than 1000 seconds (out-of-range) were measured for one of the duplicate readings. This occurred in 10 (2.5%) measurements with the Hemochron^®^ Response and in 9 (2.2%) measurements with the Hemochron^®^ Signature Elite. In the Hemochron^®^ Response also 27 (6%) “unstable magnet” errors occurred. No measurement errors were encountered with the Hemochron® Signature Elite.

**Figure 1.**
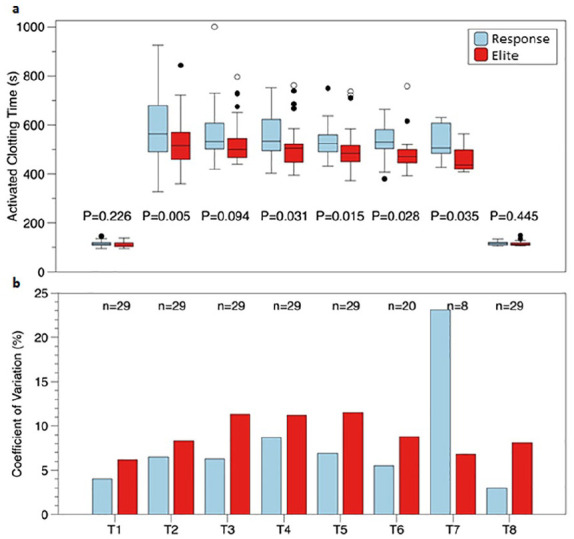
Presentation of the activated clotting time at several time points during cardiac surgery: (a) Data are presented as medians of the separate measurements with their interquartile range (IQR). Outliers (1.5 × IQR) are represented by closed circles, extreme values (3 × IQR) are represented by open circles. (b) A coefficient of variation is plotted for each time point of the two devices. The number of patients declines as the bypass time increases. *n*: number of patients at that time point.

The median ACT measurements for each device at each time point are presented in Figure 1. In the presence of heparin the Hemochron^®^ Response measurements were consistently higher over time than the Hemochron® Signature Elite measurements (p = 0.002, repeated measures). Using the duplicate averages, it was determined with a Wilcoxon signed rank test that there was a statistically significant difference between the Hemochron^^®^^ Response and the Hemochron^®^ Signature Elite after the administration of heparin, and at 30, 60, 90, and 120 minutes after the start of CPB, which is also shown in Figure 1. There was no difference in the lower ACT values (baseline and after protamine) which corresponds to the lower positive bias observed in the Bland-Altman plot. There were no statistically significant differences between the Hemochron^®^ Response duplicate measurements (p = 0.22) or between the Hemochron® Signature Elite duplicate measurements (p = 0.064). The coefficient of variation is also shown in Figure 1. This coefficient seemed to increase in the presence of heparin and was in general lower in the Hemochron® Response.

**Figure 2.**
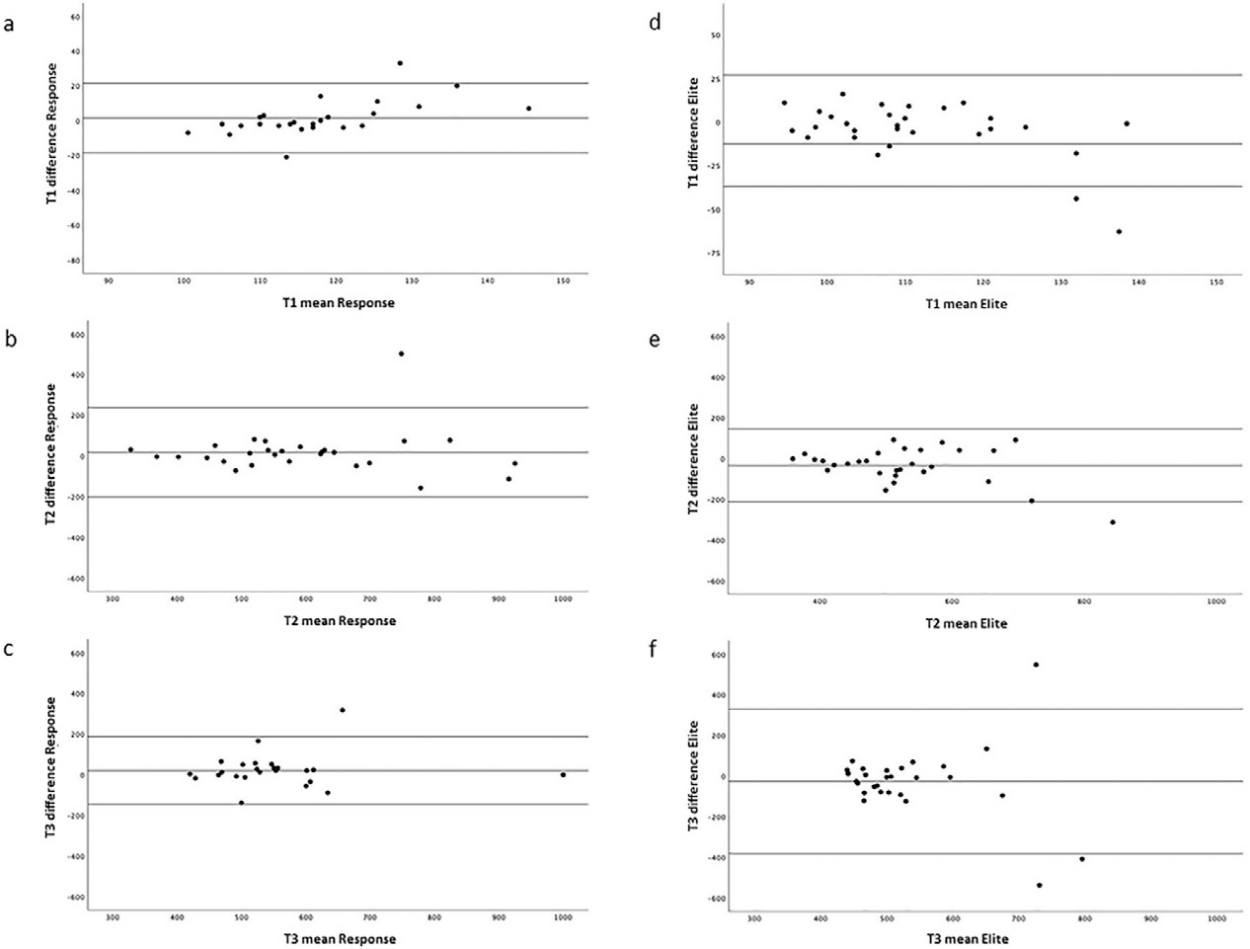
Bland-Altman plots of ACT measurements in 29 patients with the limits of agreement between the duplicate measurements of the Hemochron^®^ Response (left panel, (a)–(c)) and the Hemochron^®^ Signature Elite (right panel, (d)–(f)) at baseline (*T*1), after heparin (*T*2) and 5 minutes after the start of cardiopulmonary bypass (*T*3).

**Figure 3.**
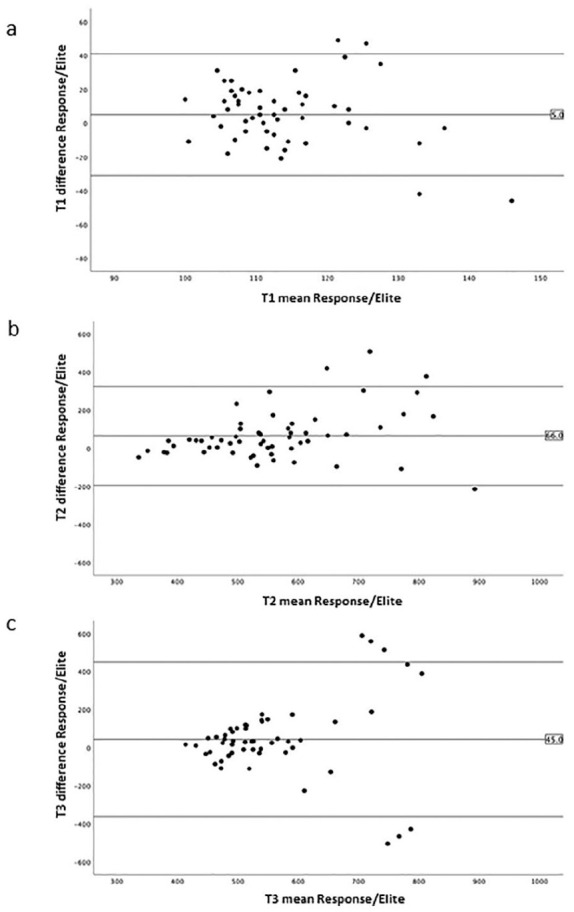
Bland-Altman plots of ACT measurements in 29 patients with the limits of agreement between the averages of the duplicate measurements between the Hemochron^®^ Response and the Hemochron^®^ Signature Elite at baseline (*T*1, (a)), after heparin (*T*2, (b)) and 5 minutes after the start of cardiopulmonary bypass (*T*3, 3(c)).

The Bland-Altman plots in Figure 2 show the limits of agreement between the duplicate measurements for both devices. These limits were slightly wider with the Elite compared to the Response. In Figure 3 a Bland-Altman plot for the same three time points is presented, but now for the average of the duplicates between both devices. The positive bias is around 5 seconds at baseline (*T*1, Figure 3(a)), 66 seconds after administration of heparin (*T*2, Figure 3(b)), and 45 seconds at 5 minutes after start of CPB (*T*3, Figure 3(c)), which are indicated in the plots and reflect the average of the differences calculated between the two method means. Positive bias values for the other time points during heparinization were 52 seconds (*T*4), 23 seconds (*T*5), 44 seconds (*T*6), and 104 seconds (*T*7) respectively, and 2 seconds after protamine administration (*T*8).

## Discussion

In this study the variability, reliability, and limits of agreement (bias) of the Hemochron^^®^^ Response were compared to the Hemochron^®^ Signature Elite during CPB in CABG or AVR patients. The reason to include both CABG and AVR patients was to introduce the same diversity in the patient population in terms of medication use as is seen in normal clinical practice. As was mentioned in the introduction, there are only two studies available that compare bias and variability in high heparin dosages such as in cardiac surgery between the Hemochron^®^ Jr ACT systems and older devices with single activators like celite or kaolin.^14,15^ Aylsworth et al.^15^ using single measurements, showed that the Hemochron^®^ Jr produced ACT values that were on average lower than the values produced by their standard the Hemochron^®^ 801. This effect was also seen for the Actalyke^®^ in comparison with the Hemochron^®^ Response, where the Actalyke^®^ was associated with lower ACT results.^17^ Lower ACT values can lead to increased heparin dosing during CPB, which may also result in excessive bleeding after cardiac surgery.^18–20^ In the presence of heparin the celite activated Hemochron^®^ Response had higher ACT values, but without heparin, that is, before and after CPB, there was no statistically significant difference between the devices. For both single kaolin measurements as well as single celite measurements a positive bias of around 86–102 seconds for the Response compared to the Hemochron^®^ Jr systems has been reported.^14,15,21^ These findings are in line with our results, which show that the Hemochron^®^ Response produces, on average, higher ACT values, especially during the heparinization period (*T*2 → T7). In contrast, lower ACT values were reported for the celite measurements compared to one of the Hemochron^®^ Jr systems in two studies during cardiac interventions. In these studies less heparin was used with lower target ACT values.^22,23^ The differences may either be caused by the use of low range cuvettes or by the computation algorithm which is part of the Hemochron^®^ Jr systems.

To our surprise a lower coefficient of variation was found in the Hemochron^®^ Response, suggesting better precision compared to the Hemochron^®^ Signature Elite. The outlier in the coefficient of variation that is seen at *T*7 with the Hemochron^®^ Response can be explained through the small number of patients (*n* = 8) left on CPB at *T*7. In both systems the coefficient of variation increased, that is, the variability of the measurements increased in the presence of heparin, and this was more pronounced in the Hemochron^®^ Signature Elite compared to the Hemochron^®^ Response, which is remarkable given its automated design. This increase in variation in the presence of heparin has been observed previously.^14,16,24^ Svenmarker et al. compared the Hemochron^®^ Signature Elite and a kaolin operated device, the Hemotec^®^ ACT monitor, and suggested that the test results were associated with the specificity of the compounds that initiated the coagulation reaction. They also analyzed the precision and bias of these two methods, but they excluded from their analysis ACT values exceeding 15% in precision and erroneous results.^14^ In our opinion, this data should have been included. The differences observed in the current study between the Hemochron^®^ Response duplicate measurements were similar as reported before,^8,11,16^ which suggests that we performed our measurements accurately. Flom-Halvorsen et al.^25^ showed a substantial variation in celite ACT measurements, and together with Bennett and Horrow,^26^ advocated the use of duplicate measurements. The variation that we measured in the Hemochron^®^ Signature Elite also supports duplicate measurements with this device.

As shown in the results some out-of-range measurements greater than 1000 seconds were seen with both devices. After analysis of these out-of-range values it was found that they only occurred in the presence of heparin, but otherwise in a random way and were all apparently false readings. These out-of-range values therefore did not play a dominant role. However, they do appear to be responsible for the non-normal distribution of the data. These out of range values were previously explained through the method of operation of the Hemochron^®^ Response.^16^ However, this finding is now remarkable as both devices operate in a completely different way. The Hemochron^®^ Response is based on capture of a magnet in the test tube by a clot, whereas in the Hemochron^®^ Signature Elite the ACT value is calculated, based on light emitting diodes (LEDs) that detect clot formation in a narrow channel. On the other hand, the Hemochron^®^ Signature Elite is more reliable because there were no erroneous measurements such as caused by a stuck magnet. This is explained by the design of the system.

Finally, the average positive bias during heparinization of around 56 seconds for the Hemochron^®^ Response compared to the Hemochron^®^ Signature Elite in the presence of heparin, that we observed in the Bland-Altman plots, could have impact on the overall heparin management. For a similar target ACT this may lead to less heparin administration if the Hemochron^®^ Response is used as a reference.^14,15^ However, target ACT values may also be changed when one device is replaced with another.^21^ No statistically significant difference between kaolin activated coagulation and celite activated coagulation guided management has been demonstrated.^16^ A number of studies suggest that post-operative bleeding and blood transfusion requirements can be reduced by better heparin monitoring techniques during surgery, resulting in the administration of less heparin.^7,8^

As a result of CPB, hemodilution occurred during surgery. The overall dilution level was about 28%. A hemodilution greater than 25% has been shown to affect celite ACT levels.^27^ A hemodilution of 40% has been shown to affect kaolin ACT levels and also Hemochron^®^ Signature Elite levels, although the last one at a reduced rate.^14^ This may in part explain the bias between the two measurement methods. However, comparable bias was present in the sample before CPB, that is, before hemodilution occurred, and after protamine administration, indicating hemodilution played only a minor role.

In this study the temperature was allowed to drift to 35°C. At these normothermic values no statistically significant effect of temperature was seen by Matte et al.^21^ who also compared the Hemochron^®^ Response and Hemochron^®^ Signature Elite ACT values.

In conclusion, the Hemochron^®^ Signature Elite was more reliable but seemed to have higher variation in the presence of heparin compared to the Hemochron^®^ Response when measuring ACT during CPB. The observed positive bias in the Hemochron^®^ Response compared to the Hemochron^®^ Signature Elite might lead to increased heparin dosing during CPB. This requires further study.
